# Uma mirada no potencial das Ciências Sociais e Humanas para o enfrentamento dos desafios que se colocam à Saúde Coletiva

**DOI:** 10.1590/0102-311XPT189924

**Published:** 2025-07-04

**Authors:** Elaine Reis Brandão

**Affiliations:** 1 Instituto de Estudos em Saúde Coletiva, Universidade Federal do Rio de Janeiro, Rio de Janeiro, Brasil.

**Keywords:** Saúde da Mulher, Interseccionalidade, Teoria Social, Ciências Sociais, Women’s Health, Intersectionality, Social Theory, Social Sciences, Salud de la Mujer, Interseccionalidad, Teoría Social, Ciencias Sociales

## Abstract

O ensaio busca refletir sobre o enorme potencial que o legado do pensamento feminista negro como teoria social crítica possui para contribuir aos desafios teóricos, metodológicos, políticos e éticos para a Saúde Coletiva. Situando a exposição no campo das Ciências Sociais e Humanas em Saúde, busca-se analisar empiricamente tais contribuições, tendo em vista a trajetória histórica do desenvolvimento dos debates públicos sobre as políticas de atenção à saúde das mulheres, ou políticas reprodutivas, em seus deslocamentos epistemológicos, sociais e políticos, até a implementação da perspectiva da justiça reprodutiva, reivindicada como uma dimensão primordial da matriz interseccional. Ancorado em uma reflexão sobre uma certa trajetória acadêmica de pesquisa nesse campo, o ensaio lança luz para o enfrentamento ético-político das expressivas desigualdades sociais no Brasil.

## Abstract (en)

This essay seeks to reflect on the enormous potential of the legacy of black feminist thought as a critical social theory to alleviate the theoretical, methodological, political, and ethical challenges for Public Health. Focusing on Social and Human Sciences in Health seeks to empirically analyze the contributions of the field in view of the historical trajectory of the development of public debates on women’s health care or reproductive policies in their epistemological, social, and political displacements up to the implementation of the perspective of reproductive justice, claimed as a primordial dimension of the intersectional matrix. Anchored in a reflection on a certain academic trajectory of research in this field, this essay sheds light on the ethical-political confrontation of the expressive social inequalities in Brazil.

## Resumen

Este ensayo busca reflexionar sobre el gran potencial del legado del pensamiento feminista negro como teoría social crítica para contribuir a los desafíos teóricos, metodológicos, políticos y éticos para la Salud Pública. Al situar la exposición en el ámbito de las Ciencias Sociales y Humanas en Salud, se busca analizar empíricamente los aportes en vista de la trayectoria histórica del desarrollo de los debates públicos sobre las políticas de salud dirigidas a las mujeres o políticas reproductivas, en sus desplazamientos epistemológicos, sociales y políticos, hasta la implementación de la perspectiva de la justicia reproductiva, reivindicada como dimensión primordial de la matriz interseccional. Anclado en una reflexión sobre una determinada trayectoria académica de investigación en este campo, este ensayo arroja luz sobre la confrontación ético-política de las significativas desigualdades sociales en Brasil.

## Introdução

A Saúde Coletiva se funda no Brasil tematizando, inicialmente, sobre as expressivas desigualdades de classe que estruturam a sociedade brasileira e as muitas iniquidades dela decorrentes, em especial no que diz respeito à universalização de direitos sociais essenciais à manutenção da vida em um patamar de dignidade humana. Em momento político estratégico de redesenho e de recomposição das forças democráticas, em diálogo com o Estado, recém-saído de uma ditadura militar, foram muitas as conquistas e avanços que obtivemos em prol da configuração do Sistema Único de Saúde (SUS). Em trabalhos sobre o balanço dos 30 anos de constituição do SUS no país ^1^ ^,^ ^2^ ^,^ ^3^, além de celebrarmos os êxitos desse árduo percurso de luta e reivindicações dos diversos movimentos sociais e trabalhadores de saúde que dele participam, vislumbramos, também, desafios que permanecem ou se recolocam, em consonância às transformações societárias das últimas décadas, no mundo e no Brasil, igualmente. 

Pensar nossas sociedades em processo de profundas transformações estruturais e subjetivas, em suas crises, imensas contradições, instabilidades sociais e políticas, bem como mudança de paradigmas, requer, como diz Fassin ^4^, um árduo trabalho de reflexão crítica. Como ele afirma, “*a reflexão epistemológica tem implicações deontológicas*” ^4^ (p. 21). Ou seja, precisamos assumir e reconhecer o intenso entrelaçamento entre ciência e moral, pautar nossas decisões e produção científica por escolhas morais eticamente comprometidas com a radicalidade democrática e a defesa intransigente dos direitos humanos.

Críticas ao chamado “Antropoceno” e suas consequências ao planeta, mudanças nos modos de produção capitalistas de bens e serviços e nos regimes políticos que os regem, cada vez mais neoliberais, acompanhados de transformações expressivas no mundo do trabalho, alterações climáticas e ambientais, migração e deslocamentos forçados em busca de sobrevivência (conflitos armados, pauperização absoluta etc.), digitalização da vida, inteligência artificial, sociabilidade entre humanos e não humanos, para findar uma lista de impactos sociais, epistemológicos, políticos e éticos que atravessaram nossas gerações. Vivemos em um tempo de extrema precarização do trabalho e de uma crise do cuidado, resultantes de processos sócio-históricos advindos da dominação colonial escravocrata que se atualizam e são agravados, com repercussões dramáticas à reprodução da vida em sociedade, a exemplo do abandono estatal à primeira infância e do envelhecimento populacional que se amplia. No nosso caso, a aprovação, em 2016, da Proposta de Emenda à Constituição, que instaura um novo regime fiscal (PEC do Teto de Gastos Públicos), e o processo de desfinanciamento das políticas de saúde no Brasil comprometem sobremaneira os esforços de redução das desigualdades socioeconômicas e das iniquidades em saúde, desvelando como o racismo institucional, como arcabouço político-ideológico que legitima e alimenta tais disparidades, continua imbricado às nossas práticas políticas e orçamentárias.

Evidentemente, sou igualmente interpelada e não terei respostas às múltiplas questões que nos afligem como cientistas sociais, mas me inspiro nas abordagens de alguns intelectuais e ativistas que admiro, como Achille Mbembe ^5^, Judith Butler ^6^ ^,^ ^7^ ^,^ ^8^, Didier Fassin ^4^ ^,^ ^9^, João Biehl ^10^, que nos ajudam a pensar o presente e o mundo futuro que queremos. Que garantias fundamentais necessitamos como sujeitos políticos para viver em sociedade existências que não sejam marginais? Descartáveis? Matáveis? Como levar a sério a interdependência em sociedade e não ceder às artimanhas do individualismo como pressuposto filosófico?

Abjeção e racismo podem ser chaves analíticas importantes para resgatarmos a compreensão da exclusão social sob novas bases teóricas e ético-políticas. O que a “ontologia corporal” como uma “ontologia social”, que Butler nos apresenta, pode nos auxiliar a elucidar os mecanismos perversos de discriminação e aniquilamento de sujeitos ou corpos políticos? Nas palavras de Butler: “[uma] *nova ontologia corporal que implique repensar a precariedade, a vulnerabilidade, a dor, a interdependência, a exposição, a subsistência corporal, o desejo, o trabalho e as reivindicações sobre a linguagem e o pertencimento social*” ^7^ (p. 15). Postulo que não há uma forma de compreendermos e incidirmos social e politicamente no mundo apartado de nossa experiência corporal (sempre visceral). Aprendemos nos movimentos feministas que a experiência corporificada engendra uma visão de mundo que nos interpela e nos posiciona diferencialmente conforme os marcadores sociais de classe, raça/etnia, gênero, geração, nacionalidade, entre outros - os chamados “conhecimento situado” ^11^ ou “conhecimento de resistência” ^12^. Se considerarmos o trabalho extraordinário de Achille Mbembe ^5^, por meio do conceito de “necropolítica”, ou de Lélia Gonzalez ^13^, com sua proposição teórica de “amefricanidade”, perceberemos como a violência e a desumanização de corpos/sujeitos estão entranhadas na dominação colonial e em suas formas mais contemporâneas, o que nos deixa marcas severas que reverberam até hoje. Voltarei, adiante, a abordar esse componente na discussão do tema da reprodução, propriamente dito.

O ensaio busca refletir sobre o enorme potencial que o legado do pensamento feminista negro como teoria social crítica possui para contribuir aos desafios teóricos, metodológicos, políticos e éticos para a Saúde Coletiva. Inspiro-me em Lélia Gonzalez ^13^, Patrícia Hill Collins ^12^, Kimberle Crenshaw ^14^, Loreta J. Ross ^15^, Mara V. Vigoya ^16^, Angela Davis ^17^, entre tantas outras intelectuais feministas negras, que enfrentaram os cânones ocidentais, produzindo clivagens teóricas e ético-políticas afrocentradas. Situando a exposição no campo das ciências sociais e humanas em saúde, busca-se analisar empiricamente tais contribuições, tendo em vista a trajetória histórica do desenvolvimento dos debates públicos sobre as políticas de atenção à saúde das mulheres, ou políticas reprodutivas, em seus deslocamentos epistemológicos, sociais e políticos, até a implementação da perspectiva da justiça reprodutiva, reivindicada como uma dimensão primordial da matriz interseccional.

Para cumprir o exercício que inicialmente me propus, como docente e pesquisadora da área das Ciências Sociais e Humanas em Saúde (CSHS), de refletir sobre a sociedade em que vivemos, sobre as ferramentas analíticas e políticas que acumulamos nas últimas décadas para compreensão e intervenção junto a nossos “objetos” de estudo e, finalmente, sobre os desafios que nos restam nos próximos anos, farei um recorte bem delimitado para tal reflexão epistêmica. Realizar tal incursão em um campo de estudos empíricos que circunda o tema da reprodução, em que construí minha trajetória de estudos, de pesquisa e de formação de novos quadros de profissionais de saúde e de pesquisadores, nos ajudará a balizar tais contribuições de modo mais claro e objetivo.

Antes, no entanto, de passarmos à digressão que pretendo trazer como exemplo para o balanço de nossa área temática no âmbito da Saúde Coletiva, gostaria de recuperar algumas ideias sobre a trajetória das CSHS no interior do campo interdisciplinar da Saúde Coletiva, e as tensões que decorrem dessa posicionalidade, no conjunto das ciências biomédicas e epidemiológicas, com as quais dialogamos e disputamos autoridade e legitimidade científica. Russo & Carrara ^18^ discutem as tensões internas ao campo da Saúde Coletiva ao abordarem duas vertentes que convivem entre si e estão entrelaçadas na história desse campo científico: uma mais acadêmica, propriamente dita, e outra mais política, voltada à formulação de políticas públicas de saúde. Parece-me que as transformações epistêmicas que estamos assistindo recentemente no interior das Ciências Sociais e Humanas nos facultam uma produção científica mais engajada, não apartada do mundo sobre o qual se reflete, comprometida social e politicamente com uma certa perspectiva de emancipação política dos sujeitos. Retomarei o exemplo de uma “epistemologia feminista negra” para submetermos tal assertiva ao escrutínio analítico. O aprendizado da interseccionalidade como ferramenta analítica e como práxis crítica e política ^19^ ^,^ ^20^ pode iluminar essa conjunção de esforços acadêmicos e de transformação social, em concomitância.

Ao recuperar as ênfases teóricas na construção social dessa área que chamamos de CSHS, Russo & Carrara ^18^ (p. 475) afirmam: “*as ciências sociais têm duas entradas no campo da SC* [Saúde Coletiva]*. Em um primeiro momento, através de uma sociologia de corte marxista e da ciência política, em que o sanitarismo predomina. Em um segundo momento, através de uma perspectiva socioantropológica, que traz consigo uma nova leitura política e representa uma vertente crítica da própria prática médica e sanitária*”. Muitas cientistas sociais de nossa geração (formação de graduação nos anos 1980) também percorreram tal caminho, no sentido de nos abrirmos a outras abordagens mais compreensivas que o materialismo histórico e dialético, que sempre continuará nos inspirando, mas sob outras elaborações e formulações teóricas mais contemporâneas, a partir, por exemplo, dos trabalhos de Michel Foucault ^21^ e tantos outros que dele derivaram suas formulações. Continuando com Russo & Carrara ^18^ (p. 479-80): “*Os novos objetos e abordagens que surgem no campo apontam para a possibilidade de trabalharmos com a ideia de uma natureza/biologia mutáveis e transformáveis em articulação com a esfera cultural/social. A transformação cultural/social, neste sentido, produziria transformações no nível material/biológico, como diversos estudos têm discutido. Ao mesmo tempo, a cultura só poderia ser pensada de forma incorporada, ou seja, existindo através do corpo, da materialidade biológica*”. Essa “virada ontológica” é crucial para nos situarmos no diálogo com novas teorias sociais críticas que nos influenciam e forjam novas epistemologias na contemporaneidade. Em ensaio sobre os desafios das Ciências Sociais e Humanas na Saúde Coletiva, Martin & Pereira ^22^ também clamam por uma nova postura intelectual e criativa que incorpore a crítica ao dualismo natureza-cultura, expresso na assunção, de um lado, da universalidade biológica, e, de outro, da diversidade cultural, provocando-nos a encampar a desconstrução desse corpo universal, não situado e limitado às suas marcas biológicas, bem como aos contornos de uma ciência estável que reitera e reifica certos cânones ocidentais e suas respectivas hegemonias de poder ^23^.

Ciente das dificuldades com que atuamos entre nossos pares e das inúmeras controvérsias que cercam a interdisciplinaridade ^24^ - nem sempre o diálogo e a negociação entre áreas de saberes distintas para intervenção em problemas complexos é possível -, creio fortemente que a Saúde Coletiva nos impulsiona a exercitar a perspectiva da totalidade, a estar atentos às relações sociais que mediam o processo de saúde e doença, inextricavelmente atreladas à nossa condição de sujeitos inseridos em territórios e contextos culturais específicos. Assim, embora sejamos um vasto campo de conhecimentos e práticas, com suas hierarquias disciplinares internas, princípios caros nos interligam tais como a soberania da vida, o combate às desigualdades sociais, a defesa de um ente público, da “ciência cidadã” ^25^ e da democracia como regime político a ser sempre aprimorado no diálogo com a sociedade civil. Como salientam Russo & Carrara ^18^ (p. 479), apostar no sentido mais radical da Saúde Coletiva, “*apontando o enraizamento social e cultural da prática biomédica e seus objetos (corpo, saúde e doença)*”, tem sido nosso maior compromisso intelectual.

## Da saúde da mulher à justiça reprodutiva: deslocamentos epistemológicos, sociais e políticos

O exemplo de um certo percurso intelectual no interior da Saúde Coletiva, vinculado à área temática das CSHS e aos estudos de gênero e da reprodução, pode ser uma boa porta de entrada para a reflexão sobre as muitas transformações epistêmicas que atravessamos ao longo das últimas décadas e nossos desafios mais prementes. Não se trata aqui de inventariar as políticas públicas de saúde desse período, mas de buscar identificar alguns deslocamentos, continuidades e rupturas epistêmicas ao longo dessas últimas décadas.

Iniciando pelo marco do Programa de Assistência Integral à Saúde da Mulher (PAISM), formulado há 40 anos no bojo dos debates públicos sobre a reforma sanitária e a construção social do SUS, em diálogo com ativistas feministas médicas, inseridas como gestoras nas burocracias do Estado ^26^ e sociedade civil organizada, sua proposta refletia um momento de reorganização dos espaços de controle social e de representação da sociedade civil organizada, a exemplo do Conselho Nacional dos Direitos das Mulheres (CNDM), das Conferências Nacionais de Saúde da Mulher em diálogo com o Estado.

Representando uma radical mudança de enfoque e da maneira de se abordar a “saúde da mulher”, até então focalizada como restrita ao binômio mãe-filho, ou seja, ao ciclo gravídico-puerperal, o novo desenho programático dialogava com a perspectiva da integralidade do cuidado ^27^ na assistência à saúde das mulheres em suas diferentes fases da vida, embora a ênfase na dimensão reprodutiva seja a que comumente adquire prioridade. O reconhecimento do exercício da sexualidade feminina e do direito ao prazer como uma esfera importante da existência das mulheres contrastava com a compulsoriedade da maternidade e afirmava outro princípio caro ao programa, qual seja, o respeito à autonomia sexual e reprodutiva. 

Rompendo com o lugar de simples objeto da intervenção do Estado para regulação da população e/ou de grupos pró-natalistas (Igreja Católica, militares) e anti-natalistas (entidades privadas internacionais e nacionais de controle populacional, médicos eugenistas), os movimentos feministas reivindicaram uma inflexão importante: posicionar as mulheres como sujeitos das políticas públicas de saúde que regulariam seus corpos e vida reprodutiva, reconhecendo o direito à autodeterminação sexual e reprodutiva como um valor fundamental à cidadania das mulheres ^28^.

Assim, abria-se um caminho para abordar o planejamento reprodutivo (antes nomeado como planejamento familiar) em uma perspectiva compreensiva, de escuta e diálogo sobre o exercício sexual heterossexual, a necessidade de prevenção à gravidez e às infecções sexualmente transmissíveis (IST), os métodos contraceptivos disponíveis na rede pública de serviços de saúde, suas vantagens e desvantagens, considerando serem classificados em três dimensões: de barreira (preservativos masculino e feminino, diafragma), hormonais (injetáveis, pílulas anticoncepcionais orais, contracepção de emergência) e não hormonais (DIU de cobre), além da laqueadura e da vasectomia, métodos definitivos de controle da fecundidade. No decorrer dos anos que se seguiram, passando pelo lançamento da Política Nacional de Atenção Integral à Saúde da Mulher (PNAISM) ^29^, um leque de ações programáticas relativas ao aprimoramento da assistência no pré-natal, parto e pós-parto, à humanização dos cuidados ofertados, à inclusão do tema da violência sexual e de gênero e a consequente organização de serviços de acolhimento às vítimas, até os serviços de aborto legal, prevenção ao câncer de colo do útero e de mamas, foram inúmeras conquistas e avanços obtidos, em diálogo com os grupos feministas e organizações não-governamentais (ONGs) voltadas à saúde das mulheres. Isso sem mencionar também a formulação de um programa e de uma política voltada à saúde de adolescentes e jovens, em que a dimensão da sexualidade e da vida reprodutiva ganhava atenção. O diálogo com os movimentos sociais permitiram a inclusão de uma diversidade de sujeitos políticos, como as mulheres do campo e das florestas, negras, quilombolas, indígenas, LGBTQIA+, em situação de rua, ciganas, com deficiências, entre outras, o que enriquecia, certamente, a complexidade da organização desses serviços de saúde.

Uma clivagem teórica importante na década de 1990, resultado do debate público feminista mais amplo, foi a incorporação gradativa do conceito de gênero, o qual desloca substancialmente a compreensão da relação entre natureza e cultura, rompendo com o vínculo atávico que submetia as mulheres a sua “natureza feminina”, essencializada e refém da reprodução. A discussão da subordinação feminina ao masculino, das hierarquias de gênero, iluminou o componente social e cultural dessa construção social, com contornos específicos em cada contexto local. Esse giro teórico e político repercute em grande escala, especialmente nas Conferências Internacionais da Organização das Nações Unidas (ONU), na década de 1990, não sem dissensos e controvérsias, nos legando as formulações de saúde reprodutiva, direitos reprodutivos e, em seguida, direitos sexuais. Não se pode dizer que incluir os direitos sexuais e os direitos reprodutivos como direitos humanos nos marcos normativos dos países signatários da Conferência Internacional de População e Desenvolvimento (Cairo, 1994), e da IV Conferência Mundial da Mulher (Beijing, 1995), seja algo relativamente simples e de pouca importância. Ainda empreendemos, tantas décadas depois, uma árdua luta e mobilização para que o direito ao aborto, de forma segura e com amparo do SUS, seja um direito das mulheres, das pessoas com útero e adolescentes brasileiras. Mas, certamente, tem sido um caminho trilhado por muitas mulheres, parlamentares, juristas, acadêmicas, profissionais de saúde, lideranças comunitárias, ativistas entre outras que costuram o cotidiano da expansão dos direitos das mulheres, na esfera do mercado de trabalho, do combate à violência de gênero, à violência obstétrica, enfrentando os retrocessos que nos atingem em razão do acirramento do conservadorismo religioso e das forças políticas extremistas. 

O tema da sexualidade, *per se*, já havia ocupado o centro do espaço público em razão da epidemia de HIV/aids, passando a ser um tema legítimo de pesquisa, de escrutínio público e de intervenções de organizações multilaterais e de saúde, tornando a discussão das políticas sexuais mais próximas do público em geral. O deslocamento e ruptura da tríade corpo-sexo-gênero (e suas expressões no desejo, atração, orientação sexual), operada primordialmente pelos trabalhos de Judith Butler ^8^, dá lugar a uma diversidade sexual e de gênero sem precedentes, na medida em que rompemos com o binarismo de gênero e com a norma heterocentrada de corpos cis. Reconhecer as pessoas trans como sujeitos políticos como outros aprofunda a máxima da autora de que sim, os corpos importam e muito. Problematizar o gênero de forma não determinada por corpos sexualmente diferentes e reintroduzi-lo no campo da linguagem, das performances discursivas no interior de cada cultura, operou outra grande clivagem teórica e política, ampliando nossa maneira de compreender as correlações possíveis entre corpo-sexo-gênero como formas subjetivas e políticas de estarmos no mundo.

Esse processo de incorporação de novas categorias analíticas à formulação de políticas públicas nunca é linear, posto que possui raízes históricas, socioculturais e políticas. Várias análises das políticas públicas voltadas à atenção à saúde das mulheres, nos anos 2000 em diante ^30^ ^,^ ^31^ ^,^ ^32^, têm destacado uma inflexão teórico-política no governo da ex-presidenta Dilma Rousseff (a partir de 2010), com retrocessos e retorno ao materno-infantilismo ^28^ ^,^ ^33^, a partir da Rede Cegonha ^34^, e da consequente centralidade da questão da gravidez, parto e pós-parto, restringindo a abordagem interseccional mais ampla que havia inspirado a PNAISM ^29^, e reafirmando a reprodução no âmbito da heteronormatividade.

Em paralelo a esses deslocamentos brevemente mencionados, é preciso resgatar outra contribuição primordial ao nosso campo da saúde reprodutiva, mas não só, fundamentalmente à saúde coletiva de forma mais ampla, qual seja, o pensamento feminista negro. Decerto, a problematização dos temas do gênero, do racismo, da interseccionalidade na saúde coletiva em contexto internacional vem sendo notada em publicações científicas de prestígio, nos últimos anos ^35^ ^,^ ^36^. A oportunidade da realização da III Conferência Mundial contra o Racismo, a Xenofobia e Intolerâncias Correlatas, da ONU, ocorrida em Durban, na África do Sul, em 2001, intensificou um processo de organização social e política dos movimentos negros, de ativistas e feministas, permitindo aprofundar a crítica ao feminismo ocidental e eurocêntrico, à dominação colonial, ao sexismo e ao racismo como ethos político vigente. 

## Pensamento feminista negro como teoria social crítica

Não precisamos ir muito longe ou buscar autoras estadunidenses, para incorporarmos a matriz interseccional como referencial analítico, para contemplarmos os fenômenos que estudamos ou lidamos no cotidiano da atenção à saúde no Brasil. A inter-relação entre os diversos marcadores sociais da diferença já estava presente sob outras nomeações/designações nas obras e textos de muitas ativistas feministas e intelectuais negras entre nós, desde muito cedo, antes mesmo do termo ser popularizado por Kimberle Crenshaw ^14^ ^,^ ^37^. Na expressão “enegrecer o feminismo”, Sueli Carneiro ^38^ já pontuava a crítica a uma matriz ocidental e branca do pensamento feminista tradicional que considerava a mulher, a princípio, como um sujeito universal, sem incorporar a dimensão das racialidades ou etnicidades. Temos orgulho em conviver e compartilhar das ideias de intelectuais negras no Brasil, como Jurema Werneck, Fatima Oliveira, Sueli Carneiro, Lucia Xavier, Lélia Gonzalez, e Mara V. Vigoya, na Colômbia, entre tantas outras que nos inspiram sempre. De modos distintos, todas nos sensibilizam sobre as muitas opressões que permeiam a existência das mulheres negras, bem como dos constrangimentos sociais por elas sofridos na interseção do gênero com seu pertencimento racial e subordinado como classe social. Desde a concepção do conceito de “amefricanidade”, trazido por Lélia Gonzalez ^13^ aos “dispositivos de racialidades” de Sueli Carneiro ^39^, somos forjadas a encarar as entranhas de nosso passado colonial, no Brasil e na América Latina, e desconstruir uma narrativa épica vitoriosa de colonização que ainda impregna nosso inconsciente coletivo. 

As contribuições do pensamento feminista decolonial ^40^ nos instauram a pensar a dominação capitalista alicerçada não somente na exploração de determinada classe social, mas também, e concomitante, na exploração de pessoas racializadas como negras e posicionadas segundo o gênero. Como afirma Patrícia Hill Collins ^12^, a interseccionalidade se apresenta como um “projeto de conhecimento” e uma “arma política”. Diante à resistência epistêmica ao tema, ela nos propõe as “comunidades interpretativas” - uma forma de abertura ao saber, à partilha, ao diálogo epistêmico como um modo diferente de produção do conhecimento como conhecemos até então. Nessa matriz teórica crítica, a experiência e o conhecimento corporificado são valorizados, assim como sua capacidade e aspiração emancipatória dos sujeitos em aliança com um *ethos* de justiça social.

Aqui reside uma chave de análise estratégica para resgatarmos uma dimensão que foi se perdendo, a meu ver, ao longo do tempo, diluindo-se em transformações tecnológicas que nem sempre traduzem o espírito que anima o sonho democrático, ou seja, a defesa intransigente da justiça social. Retomando a formulação do PAISM nos anos 1980, e do próprio SUS, esse horizonte ético-político estava lá nos inspirando, em sua origem, mas ao longo dos anos, tais princípios ou pressupostos foram reapropriados pela lógica neoliberal e se tornaram premissas de “autocuidado” e de “escolha livre e informada”, apartados de mínimas condições sociais que sustentem tais “escolhas”. A privatização do cuidado tem sido uma decorrência do avanço da agenda neoliberal, como destaca Laura Briggs ^41^. O ideário dos direitos tem sido cooptado e reduzido a estratégias sociotécnicas. O senso de justiça social subjacente à concepção de saúde consagrada na *Constituição Federal* de 1988 foi se perdendo, diluindo-se nos famigerados embates políticos e negociações entre grupos que concentram privilégios e poderes, tornando a dimensão técnica preponderante em relação à dimensão emancipatória das práticas em saúde. Várias análises críticas desse percurso sinuoso já foram realizadas, evidenciando as fragilidades que comprometiam a efetiva institucionalização das políticas de saúde e dos direitos assegurados na Constituição e na Lei de Planejamento Familiar, de 1996 ^28^ ^,^ ^42^ ^,^ ^43^.

Em outro trabalho ^44^, já havíamos comentado sobre a radicalidade das premissas que inspiraram a formulação de direitos sexuais e reprodutivos nos seus primórdios, expressa em texto seminal de Corrêa & Petchesky ^45^, contrariando a lógica individualista e neoliberal. Nessa discussão sobre o arcabouço ético, teórico, político e jurídico que circunscrevia os direitos sexuais e reprodutivos, Corrêa & Petchesky ^45^ exploram as múltiplas tensões entre os princípios da liberdade individual e da justiça social que permeiam os debates feministas há décadas. Elas destacavam quatro princípios éticos profundamente válidos atualmente: integridade corporal; autonomia pessoal; igualdade e diversidade. Defender a autonomia sexual e reprodutiva das mulheres sem condições sociais estruturais para manutenção de uma vida digna não nos permite ir muito longe. As circunstâncias sociais que balizam as decisões de vida das mulheres negras de segmentos sociais excluídos restringem, sobremaneira, suas possibilidades de escolha. 

A perspectiva interseccional nos conduz a considerar os distintos sistemas de classificação social (como classe, sexo, raça/cor, gênero, geração e nacionalidade) de modo concomitante como classificações cruzadas, que são potencializadas reiterando relações de poder e de dominação. Torna-se uma lente para ampliar a compreensão da complexidade da (re)produção das desigualdades sociais em contextos específicos. O fato de estarmos nos deparando atualmente com muito mais frequência com essas manifestações teóricas, políticas, artísticas, culturais, ativistas (*black lives matter -* vidas pretas importam), nos tira da posição de conforto como mulheres brancas, de classes médias, intelectualizadas, feministas, mas que não vivenciam a experiência da dor de uma existência negra em um país extremamente racista, com instituições racistas que aniquilam a possibilidade de alcançarmos a tão sonhada equidade social.

Temos uma enorme dívida social a saldar com a grande maioria da população brasileira se de fato aspiramos às transformações sociais estruturais para garantir segurança alimentar, moradia em territórios não violentos, educação, saneamento, entre tantas dimensões imprescindíveis a uma vida com dignidade e respeito. Mas não basta transformarmos as dimensões de gênero ou raciais em meros indicadores de saúde, como inclusões tecnocráticas assépticas que anulam a radicalidade teórica do pensamento feminista negro. Não se trata de uma luta identitária, muito menos essencialista, trata-se de um ideal emancipatório que restaura a perspectiva da justiça social como algo a ser perseguido por toda a sociedade.

A meu ver, a matriz interseccional nos permite refletir sobre a necessidade de um pacto civilizatório, por meio de sua práxis crítica, no enfrentamento do genocídio de pessoas negras e pobres, jovens, do encarceramento em massa, da mortalidade infantil e materna, do racismo obstétrico, da violência sexual que subjuga meninas e adolescentes, entre tantos dramas que escancaram a perversidade desse sistema social. Segundo Collins & Bilge ^46^ (p. 45), a interseccionalidade reúne “*seis ideias centrais: a desigualdade social, as relações de poder interseccionais, o contexto social, a relacionalidade* [interconexões]*, a justiça social e a complexidade*”.

“*A interseccionalidade investiga como as relações interseccionais de poder influenciam as relações sociais em sociedades marcadas pela diversidade, bem como as experiências individuais na vida cotidiana. Como ferramenta analítica, considera que as categorias de raça, classe, gênero, orientação sexual, nacionalidade, capacidade, etnia e faixa etária - entre outras - são inter-relacionadas e moldam-se mutuamente*” ^46^ (p. 16). Ainda apoiada em Collins & Bilge ^46^ (p. 16), “*a interseccionalidade é uma forma de entender e explicar a complexidade do mundo, das pessoas e das experiências humanas*”. 

“*Como as ideias e a práxis crítica da interseccionalidade estão alinhadas com o ethos dos direitos humanos, o seu uso como ferramenta analítica pode ser uma lente crítica importante para as iniciativas em favor dos direitos humanos*” ^46^ (p. 67) e da “*inclusão democrática*” ^46^ (p. 85). Vejamos, agora, como tal perspectiva teórica e política repercute na noção de justiça reprodutiva.

## O ativismo interseccional condensado na perspectiva da justiça reprodutiva

A história do surgimento da ginecologia como disciplina científica ^47^, do controle populacional sob a égide da eugenia e de suas marcas racistas no exercício das práticas médicas com ênfase na reprodução têm sido objeto de estudos históricos e antropológicos há várias décadas no Brasil ^48^ e no contexto internacional ^17^ ^,^ ^49^ ^,^ ^50^. São bastante conhecidos os episódios de esterilizações forçadas, testes clínicos em populações em situação de vulnerabilidade, tecnologias contraceptivas que produzem danos e prejuízos à saúde das mulheres, em geral, negras e pobres. A atualidade dessas análises tem sido recuperada nas investigações sobre o que as antropólogas Morgan & Roberts ^51^ chamam de “governança reprodutiva”, concebida como: “*Os mecanismos pelos quais diferentes configurações históricas de atores, como instituições estatais, religiosas e internacionais financeiras, ONGs e movimentos sociais, usam controles legislativos, incentivos econômicos, injunções morais, coerção direta e incitamentos éticos para produzir, monitorar e controlar comportamentos reprodutivos e práticas populacionais*” ^51^ (p. 243).

O conceito de governança reprodutiva não é novo, assim como as conexões entre raça, classe social, reprodução, sexualidade e saúde também não o são. Nosso desafio é elucidar as formas contemporâneas que tais estratégias sociotécnicas e políticas assumem em estreita porosidade com o discurso dos direitos humanos. Ao ressignificar o controle reprodutivo:

“*Recuperando conceitos caros a esse campo de discussão como ‘reprodução estratificada’, ou seja, acesso desigual e estratificado a condições sociais estruturais para se reproduzir, elas destacam as intricadas relações entre movimentos mais amplos, em nível global, que rearticulam interesses financeiros, hegemonias políticas, imperialismos coloniais, ativismos sociais e tecnologias biomédicas na direção de um bem-estar global que se consubstanciam de modo difuso, intersticial em práticas políticas locais, as quais consolidam ‘regimes morais de reprodução’. Desse modo, as autoras nos ajudam a complexificar os sentidos que atravessam as múltiplas tecnologias de reprodução como formas de pensar o gênero, a dimensão étnico-racial e de governar os corpos*” ^52^ (p. 52).

Recentemente, em trabalho publicado ^52^ sobre a difusão dos métodos contraceptivos reversíveis de longa duração (*long-acting reversible contraception* - LARC), no Município de São Paulo, retomamos algumas formulações que têm sido utilizadas na literatura internacional sobre o tema para qualificar formas mais sofisticadas e mais sutis, em consonância ao ideário dos direitos humanos e das mudanças ambientais, de controle reprodutivo designado por certos autores como “populacionismos” ^53^ ^,^ ^54^ ^,^ ^55^ ou “coerção contraceptiva” ^56^, em estreita articulação com os dispositivos de sexualidade ^57^ e de racialidade ^39^.

Os chamados “populacionismos” expressam um conjunto de intervenções que mantêm a premissa central de que os problemas sociais e ambientais são decorrentes do crescimento populacional. Assim, condensam um conjunto de iniciativas de intervenção global, lideradas por consórcios multilaterais que congregam ONGs, fundos internacionais, doadores privados, laboratórios farmacêuticos, agências multilaterais, associações médicas, entre outros agentes na promoção do acesso a métodos contraceptivos em países do Sul Global. Sob o argumento de expansão do acesso a novas e modernas tecnologias biomédicas contraceptivas como afirmação de um direito reprodutivo das mulheres, nem sempre os processos de trabalho e de articulações políticas, bem como as práticas de saúde dele derivadas respeitam as prerrogativas de autonomia sexual e reprodutivas dessas mulheres, em geral, pobres, negras e vivendo em contextos de extrema vulnerabilidade social. Sob o manto do pragmatismo neoliberal, tais políticas públicas de saúde relativas ao planejamento reprodutivo podem terminar institucionalizando novas formas de tutela e de controle da vida reprodutiva das mulheres pelo Estado ou parcerias público-privadas.

No legado oriundo dos feminismos negros, a perspectiva da justiça reprodutiva ^15^ ^,^ ^58^ ^,^ ^59^ ^,^ ^60^ torna-se um horizonte ético-político imperativo para lutarmos por equidade e justiça social no SUS, frente às ameaças constantes de subtração de nossos direitos. O paradigma da justiça reprodutiva compreende o direito das mulheres a não ter filhos, assegurandos-lhes o direito ao aborto seguro, o direito a ter filhos, assegurando-lhe os meios e condições para tal gestação e, principalmente, o direito a criá-los dentro de patamares da dignidade humana, ou seja, mediante condições sociais estruturais que lhes permitam viver em segurança e com apoio do Estado, por meio de políticas públicas de segurança alimentar, habitação, saneamento, educação, saúde, entre outras.

Uma das primeiras ativistas feministas negras a problematizar a acepção de justiça reprodutiva, na década de 1990, entre coletivos de mulheres negras nos Estados Unidos, foi Loretta Ross, fundadora em 1997 do grupo *SisterSong Women of Color Reproductive Justice Collective* (Coletivo de Justiça Reprodutiva de Mulheres de Cor da SisterSong). Hoje o conceito tem sido amplamente discutido no meio acadêmico, jurídico e ativista. Adotando a teoria feminista negra em sua radicalidade para abordar questões reprodutivas, essa concepção produz uma crítica à perspectiva liberal centrada no par “*pro-choice/pro-life*”, que tematizou a luta pelo direito ao aborto nos Estados Unidos, produzindo outra práxis política. Ao apontar limites dessa concepção mais estreita, que embasa o direito à escolha reprodutiva, não tomando a maternidade como compulsória - ter ou não ter filhos -, o conceito de justiça reprodutiva, cunhado em 1994, abarca também o direito a ter filhos em condições seguras, independente da condição social das mulheres (privadas de liberdade, em situação de rua, em abrigos). Como enfatiza Dorothy E. Roberts ^60^ (p. 79): “*é um paradigma que inclui não apenas o direito da mulher de não ter filhos, mas também o direito de ter filhos e de criá-los com dignidade em ambientes seguros, saudáveis e de apoio*”. Em síntese, não se pode falar em autonomia (autodeterminação) reprodutiva quando há obstáculos estruturais que impedem tais escolhas, ou ainda, que perpetuam a desigualdade social, racial, de gênero. Tal compreensão implica no descentramento da responsabilidade das próprias mulheres por sua (in)capacidade de gerir sua vida sexual e reprodutiva, para obrigar o Estado a ampará-las em algo que alude à reprodução da vida em sociedade. Nesse sentido, não podemos reiterar que as “escolhas” sejam individuais, tendo em vista a mercantilização da reprodução ^59^.

## Planejamento reprodutivo: desafios que se atualizam

Assim como nós, pesquisadoras sobre o tema da reprodução, na Saúde Coletiva ou em outros campos disciplinares, fomos interpeladas por tais críticas contundentes e instadas a incorporar as matrizes da interseccionalidade e da justiça reprodutiva em nossas investigações empíricas, no ensino, na nossa produção científica, acredito seriamente que a Saúde Coletiva só terá a ganhar com tal abordagem teórica e política. Adotar a perspectiva feminista negra interseccional para pensar o país e suas imensas dívidas sociais, rompendo definitivamente com nossa matriz colonial e racista, precisa ser um compromisso assumido por todos nós, que combatemos as desigualdades sociais. Ao reconhecermos o protagonismo dos movimentos sociais feministas negros, das mulheres e homens negros usuários dos serviços públicos de saúde, contemplados pela cobertura universal do SUS, uma subversão do conhecimento se produz, permeável aos saberes outros. Tal postura ética e política pode radicalizar a maneira como pensamos nossos temas de pesquisa e modos de atuação na Saúde Coletiva e na vida universitária. Assim, seria possível produzir em aliança com tais sujeitos políticos um conhecimento engajado, não à distância, mas prenhe de valores e de compromisso ético e político com a transformação social de tantas injustiças. Quem sabe poderíamos, inspiradas nas formulações de uma “ética autorreflexiva”, como sugere Patricia Hill Collins ^12^, aliada a uma “ética do cuidado”, na acepção de João Biehl ^10^, apostar em uma resistência epistêmica que consolide nossas raízes ancestrais indígenas e africanas?

O exemplo de algumas pesquisas realizadas nos últimos anos, amparada nesse referencial, pode ajudar na compreensão da validade teórica e metodológica de tal perspectiva. Sob o escopo da “governança reprodutiva”, antes mencionada, tenho me debruçado sobre alguns objetos mais específicos para pensar o problema da reprodução entre nós, no sentido de um desafio muito peculiar ao cotidiano de jovens e mulheres - evitar a gravidez. São eles, os contraceptivos de emergência ^61^; os LARC ^52^ ^,^ ^62^ e o dispositivo de esterilização Essure ^63^ ^,^ ^64^ ^,^ ^65^.

Em poucas palavras, busco sintetizar algumas análises empreendidas nessas investigações. No tocante à contracepção de emergência, observamos uma expansão significativa de sua utilização nas gerações mais jovens, com recurso predominante às farmácias comerciais (usualmente pelos parceiros) e não ao SUS, por desejo de discrição e privacidade, para não se submeter a julgamentos morais por parte dos profissionais de saúde e pela celeridade da compra e uso imediato do medicamento, o que importa nesse caso. Como estamos com uma defasagem imensa de dados sociodemográficos sobre práticas contraceptivas, em razão da *Pesquisa Nacional de Demografia e Saúde* (PNDS) não ter sido realizada em 2016 até hoje, há um vácuo imenso na mensuração desse recurso. O maior problema é o silenciamento público sobre o tema nas campanhas educativas de prevenção à gravidez, na difusão dessas informações que são importantes e podem ajudar a prevenir uma gravidez. Há muitas reticências por parte das equipes de saúde em divulgar tal alternativa, embora seja um direito assegurado às usuárias.

No tocante aos LARC, percebemos exatamente o contrário, principalmente em relação aos LARC hormonais. Muita propaganda, divulgação midiática, incentivo das empresas farmacêuticas e associações médicas, um *lobby* poderoso que tenta nos convencer das maravilhas desses métodos. A cautela é sempre necessária diante a tanto entusiasmo, pois geralmente tais estratégias penetram no Estado mobilizando recursos vultuosos, com certas direções, ou seja, são comumente endereçadas prioritariamente às mulheres em situação de vulnerabilidade social, sejam abrigadas, em situação de rua, usuárias de substâncias, trabalhadoras do sexo, adolescentes etc. Enfim, a oferta não costuma ser universal e ampla a todas as usuárias que desejarem. E também nos preocupamos com o período pós-inserção, quando a vida com o método acontece e seus efeitos colaterais podem surgir. Em geral, as metas institucionais são destinadas a contabilizar os procedimentos de inserção, relegando o acompanhamento clínico posterior e eventualmente sua retirada antes do prazo expirar. Decerto, os LARC podem ser boas alternativas para ampliar o leque de possibilidades às usuárias, mas não podem ser tomados como panaceia e solução a todas indistintamente.

Quanto ao dispositivo de esterilização permanente Essure, fabricado pela Bayer, e já retirado do mercado pela própria empresa, tenho acompanhado mais de perto a trajetória do grupo de mulheres do Rio de Janeiro que aguardava para fazer a ligadura de trompas e foram convocadas para receber o dispositivo no Hospital Municipal Mariska Ribeiro, em Bangu, no período que ele circulou no Brasil, de 2009 a 2017. O procedimento prometido seria inócuo, rápido e simples, com realização ambulatorial e deveria protegê-las da gravidez. Infelizmente, não foi o que ocorreu, além de gravidez, surgiram também muitos outros problemas de saúde decorrentes do dispositivo. Com a interrupção de sua circulação no mercado, tais usuárias ficaram desamparadas no tocante à assistência a seus problemas de saúde. Em resumo, elas se reuniram via redes sociais, criaram estratégias de comunicação e de sensibilização pública, convocando parlamentares, autoridades de saúde, defensoria pública, no intuito de lutarem pela retirada do dispositivo de seus corpos, por via cirúrgica, o que torna mais complexo os procedimentos médicos necessários. Há grupos em Brasília, São Paulo e no Rio de Janeiro em busca de justiça e reparação pelos danos causados pelo dispositivo. Esse é mais um exemplo do modo como novas tecnologias biomédicas são testadas ou aprimoradas em corpos de mulheres periféricas, como Rosa Germano, do grupo “Vítimas do Essure”, no Rio de Janeiro, se expressa, sem o devido comprometimento ético com a segurança e saúde das usuárias.

Assim, as estratégias de governança reprodutiva podem ser dissecadas e enfrentadas em uma práxis política e crítica com apoio nas abordagens interseccionais. Ao lançar mão da contracepção de emergência, após uma relação sexual desprotegida, enfrentando discriminações morais e preconceitos sociais, sendo antecipadamente recriminada individualmente pela “desproteção” (de origens diversas) de um ato relacional (com o parceiro), elas afirmam sua autonomia e reivindicam um direito que lhes assegure, após o ato sexual, certa proteção à gravidez. As usuárias que demandam os métodos contraceptivos reversíveis de longa duração - hormonais ou não - como o dispositivo intrauterino (DIU) de cobre ou os implantes subdérmicos e sistema intrauterino (SIU) hormonal, estão buscando fugir dos terríveis efeitos colaterais dos métodos hormonais de uso regular ou diário, como as pílulas ou injetáveis, minimizando assim seus mal-estares e riscos do uso contínuo de hormônios em sua saúde. Por outro lado, as usuárias do dispositivo permanente de esterilização Essure, que sofrem com seus efeitos colaterais e problemas de saúde dele derivados, estão encampando uma mobilização social sem precedentes junto ao Estado e ao poder médico, na busca por justiça social e por reparação pelos danos obtidos em sua vida, ao reivindicarem a retirada do dispositivo de seus corpos e cuidados de saúde para tal acompanhamento clínico. Na tessitura entre estratégias biopolíticas e afirmação de desejos e autonomia sexual e reprodutiva, as mulheres vão tecendo suas escolhas e identificando possibilidades de garantia de direitos.

As usuárias do SUS - que sempre acompanhei ao longo de tantas décadas analisando os desafios da contracepção e da gravidez imprevista em um país que interdita o aborto seguro como um direito das mulheres cis e das pessoas com útero, sistematicamente negras, pobres, residentes em territórios periféricos - têm encontrado umas às outras nas redes sociais, buscado garantir seus direitos sexuais e reprodutivos, identificando-se com pautas emancipatórias e campanhas de combate à violência sexual e de gênero, ao racismo obstétrico, apoiado outras mulheres, rompendo gradativamente com estruturas rígidas e hierárquicas de poder. De certa forma, a duras penas elas encontram formas de resistir e de agenciar suas necessidades e desejos em contextos adversos, renovando nossas esperanças de que o acolhimento e a atenção ou cuidado em temas tão íntimos e delicados deva ser primordialmente ético e respeitoso. Respeitar a autonomia sexual e reprodutiva de todas essas mulheres é um princípio caro que nos move.

Ao refletir sobre os nossos desafios mais prementes, considero que incorporar no ensino e na pesquisa, na formação de profissionais de saúde, essa matriz social crítica interseccional, derivada do pensamento feminista negro, seria nossa maior prioridade nesse momento. Assim como incluir nos espaços de formação e de exercício profissional o tema da justiça reprodutiva. Incorporar as dimensões macrossocietárias relativas ao gênero, às racialidades, às classes e ao ethos científico feminista são conteúdos fundamentais para que estudantes possam fazer escolhas éticas e democráticas, atinentes à defesa dos direitos humanos. Não fazer concessões, abrir espaço para nossos estudantes terem voz, formularem uma reflexão teórica e crítica engajada com o contexto sociocultural que vivemos, respeitando nossas raízes ancestrais, a diversidade de culturas que convivem no Brasil, povos quilombolas, ribeirinhos, indígenas, distintas crenças religiosas, expressões de gênero. Precisamos aprender a construir pontes, dialogar com nossos estudantes, apostar em seu potencial como sujeitos capazes de atravessar as muitas precariedades que nos cercam e fomentar a transformação desse cenário. Nossos discentes precisam se ver representados no corpo docente das universidades, para terem esperanças de voar alto, ser quem eles quiserem ser... Introduzir em nossos programas de curso, nos conteúdos didáticos que ministramos autores e temas diversos que nos instrumentalizem na luta antirracista, anticapacitista, antissexista, não LGBTQIA+fóbica, nos ajudará a trazer o cotidiano vivido e suas marcas de desumanização para reflexão na sala de aula. Só assim, creio, fará sentido para nossos jovens estudantes ter um conhecimento que dialogue com suas experiências de vida e com seu entorno territorial.
